# A modified method of separating Tl(I) and Tl(III) in aqueous samples using solid phase extraction

**DOI:** 10.1186/s13065-018-0502-6

**Published:** 2018-12-05

**Authors:** Qingxiang Xiao, Atta Rasool, Tangfu Xiao, Philippe C. Baveye

**Affiliations:** 1 State Key Laboratory of Environmental Geochemistry, Institute of Geochemistry, Chinese Academy of Sciences, Guiyang, 550081 China; 2 Key Laboratory for Water Quality and Conservation of the Pearl River Delta, Ministry of Education, School of Environmental Science and Engineering, Guangzhou University, Guangzhou, 510006 China; 3 University of Chinese Academy of Sciences, Beijing, 100049 China; 4 UMR Ecosys, Université Paris-Saclay, AgroParisTech, Avenue Lucien Brétignières, 78850 Thiverval-Grignon, France

**Keywords:** Thallium (III), Speciation, SPE, AG1-X8

## Abstract

In spite of the development of new measurement techniques in recent years, the rapid and accurate speciation of thallium in environmental aqueous samples remains a challenge. In this context, a novel method of solid phase extraction (SPE), involving the anion exchange resin AG1-X8, is proposed to separate Tl(I) and Tl(III). In the presence of diethylene triamine pentacetate acid (DTPA), Tl(III) and Tl(I) can be separated by selective adsorption of Tl(III)-DTPA onto the resin, Tl(III) is then eluted by a solution of HCl with SO_2_. The validity of this method was confirmed by assays of standard solutions of Tl(I) and Tl(III). The proposed method is shown to have an outstanding performance even in solutions with a high ratio of Tl(I)/Tl(III), and can be applied to aqueous samples with a high concentration of other electrolytes, which could interfere with the measurement. Portable equipment and reagents make it possible to use the proposed method routinely in the field.

**Electronic supplementary material:**

The online version of this article (10.1186/s13065-018-0502-6) contains supplementary material, which is available to authorized users.

## Introduction

Thallium (Tl), a toxic trace metal and one of the USEPA’s priority metal pollutants [1]. Although the abundance of Tl is low in the Earth’s crust (generally < 1 mg/kg), Tl contamination is increasing worldwide due to metal mining/smelting activities as Tl is abundant in many hydrothermal sulfide deposits [2]. Serious Tl pollution has been reported in many environmental matrices collected from the mining areas [3–5]. Thallium concentrations in uncontaminated waters are usually low, generally below < 1–2 μg/L [2]. However, at sites that are influenced by mining activities, high levels of Tl in waters have been reported [6–18].

In nature, Tl mainly exists in two oxidation states, Tl(I) and Tl(III) [19, 20]. The different chemical forms have marked different toxicities, mobilities, and biological activities. For example, Tl(III) is about 50,000 times more toxic than Tl(I) to alga *Chlorella* as the free ion [21]. Therefore, it is important to determine which form of Tl is present. In recent years this has led to a significant effort to develop methods for the speciation of Tl in environmental samples [19]. The main analytical methods used to separate and pre-concentrate Tl species include solid phase extraction [22–31] and liquid chromatography [6, 19, 32–37]. Different chromatographic techniques, such as cation exchange [6, 33, 38], anion exchange [32, 33, 36, 37], size-exclusion chromatography (SEC) [33], and reversed phase-chromatography [35] have been used to measure the redox state of Tl(I) and Tl(III) in natural samples, with the use of inductively coupled plasma mass spectrometry (ICP-MS) as the mass selective detector. The chromatographic techniques have the advantage of automatic operation, good sensitivity and separation efficiency, however, the rigorous experimental conditions required have deterred many researchers. Moreover, as Tl(III) is usually at trace levels it must be pre-concentrated prior to measurement, making the chromatographic technique difficult. In this regard, solid phase extraction (SPE) can separate and pre-concentrate Tl(III), making the technique attractive for in field preservation or measurement [25]. Samples prepared by SPE can be preserved for a long time prior to analysis. The portable nature of the experimental equipment required for SPE make it a good candidate for Tl speciation measurement in the field.

Speciation computational studies of the speciation of Tl have provided some insights into the redox state of Tl in natural waters. According to these computation studies, approximately 10% of Tl(I) in water can be present as a TlSO_4_^−^ complex when SO_4_^−^ is increased to 10^−2^ mol/L [39]. Tl(III) in water is likely to precipitate as Tl(OH)_3_ at pH < 7 (Ksp25 °C = 1.68 × 10^−44^), whereas Tl(OH)_2_^+^ and Tl(OH)_2_^−^ are likely to form at even lower pH of 2 [6, 32]. The most interesting prediction of speciation computations is that reduction of Tl(III) to Tl(I) is spontaneous due to a high reduction potential (+ 1.26 V), unless stable complexes are formed, such as TlCl_4_^−^ (logK = 18), Tl(III)-EDTA (logK = 22.5), Tl(III)-DTPA (logK = 46), or Tl(III)-DDTC will precipitate [22]. Therefore, the addition of a complexing agent is necessary to preserve Tl(III) in any given aqueous system and prevent reduction to Tl(I). A number of studies have used Cl^−^ and DDTC as complexing agents for the determination of Tl(III) species in water samples [20, 40, 41]. However, diethylene triamine pentoacetic acid (DTPA) is more commonly used for Tl speciation as DTPA has a higher stability constant than Cl^−^, and it does not precipitate like DDTC. In addition, the Tl(III)-DTPA complexes is stable during chemical preparation processes, and is stable for 7–10 days after preparation even when exposed to UV radiation [42]. More importantly, Tl(I) does not form a complex with DTPA [6]. To date, however, few studies have used DTPA to speciation of Tl, mostly in tandem with liquid chromatography [33, 36–38], and SPE [25, 31], making it a good candidate to modify for field measurements. The use of alumina by some reported methods [25, 31] could pose potential issues as it can absorb both the cation Tl(I) and the anionic group of Tl(III)-DTPA [43], which makes finding a stable and accessible sorbent difficult. The anion exchange resin AG1-X8 has been used to pre-concentrate Tl for Tl isotope measurements [44]. Batley and Florence [20] used AG1-X8 to pre-concentrate Tl and assess the concentrations of Tl(I) and Tl(III) in seawater. However, it is not clear how applicable the method to non-saline samples that differ substantially from the seawater for which it was developed.

Thus, the objective of the research presented in this manuscript is to combine the use of the AG1-X8 resin for the separation of Tl(I) and Tl(III) with DTPA used as the complexing agent. In principle, this approach should enable the pre-concentration of trace levels of Tl(III) and make it possible to quantitatively determine Tl(I) and Tl(III) in wastewater in the presence of high concentrations of potentially interfering ions. Another advantage of this approach is that it should be simple to implement under field conditions, as separation of Tl(I) and Tl(III) is performed in the field via the addition of DTPA and separation by the SPE resin prior to measurement by ICP-MS.

## Experimental

### Reagents

Type 1 water (> 18.2 MΩ-cm) was used throughout the experiment for the preparation of all reagents and standards. AG1-X8 analytical-grade anion-exchange resin was purchased from BIO-RAD (http://www.bio-rad.com) in the chloride form with a dry mesh size of 200–400 and a wet bead size of 45–106 μm, and a nominal capacity of 1.2 meq per mL of resin bed. Prior to use, the resin was washed with solutions of NaCl, NaOH, and HCl in sequence to remove any organic and inorganic impurities according to the following steps. (1) AG1-X8 resin is washed with a saturated sodium chloride solution in a mass to volume ratio of V_aq_(NaCl)/V(resin) = 3:1 and then placed into a separating funnel. After allowing to soaking for 24 h, the solution was discharged, and the resin was washed three times with type 1 water; (2) a 1 mol/L sodium hydroxide solution is added to the resin in a beaker in the same proportions as previously (V_aq_(NaOH)/V(resin) = 3:1) and allowed to stand for 8 h, it is then separated and washed as previously; 3) 1 mol/L Hydrochloric acid (1 mol/L) is added in the same ratio (V_aq_(HCl)/V(resin) = 3:1) and allowed to stand for 8 h and washed as previously. The resin is then preserved in a 0.1 mol/L HCl solution for later use.

DTPA (analytical reagent, ≥ 99%) was purchased from Sigma-Aldrich. The solution of DTPA (10 mmol/L) was obtained by dissolving 3.93 g DTPA in 1 L type 1 water (> 18.2 MΩ-cm) and heated to 373 K for approximately 20 min until dissolved. The solution was allowed to cool to room temperature prior to use. Standard solutions of Tl(I) and Tl(III) were prepared when needed by dissolving TlNO_3_ (Sigma-Aldrich) in 0.5 mmol/L HNO_3_ or by dissolving Tl(NO_3_)_3_·3H_2_O (Sigma-Aldrich) in 10 mmol/L DTPA + 5 mmol HNO_3_ solution and diluting to the desired concentration with 400 mg/L. The standard solution of Tl(III) obtained from Tl(NO_3_)_3_·3H_2_O was subsequently oxidized via the following procedure. (1) 0.5 mL of saturated bromine water was added to 10 mL of the Tl(III) solution and stirred for 15 min with a glass rod. (2) 10 mL of a 10 mmol/L DTPA solution was then added, and the solution was stirred for 15 min, then heating for a further 30 min until colorless, the volume is adjusted to 20 mL, and the solution was placed into the dark for further use. Saturated bromine water was prepared when needed by adding 1 mL bromine (99.99% metals basis) to 20 mL water. The SPE eluent containing 5–6% SO_2_ in 0.1 M HCl (w/w; hereafter abbreviated as 0.1 M HCl–SO_2_) was prepared following the procedures outline by Rehkämper and Halliday [45].

The solution of saturated NaCl and 1 mol/L NaOH were prepared by dissolving 36 g NaCl (analytical reagent, ≥ 99%) and 4 g NaOH (analytical reagent, ≥ 99%) in 100 mL type 1 water, respectively. The solutions containing 0.1, 1 and 6 mol/L HCl was prepared by adding 0.83, 8.3 and 50 mL HCl (Guaranteed reagent, 36–38%) in 100, 92 and 50 mL type 1 water, respectively. The solution of 10 mol/L HNO_3_ was prepared by adding 71.4 mL HNO_3_ (Guaranteed reagent, 65–68%) in 29 mL type 1 water. The solutions of 1 mol/L KI, 1 mol/L sodium thiosulfate and 1 mol/L sodium citrate were prepared by dissolution of 16.6 g KI (analytical reagent, ≥ 99%), 15.8 g sodium thiosulfate (analytical reagent, ≥ 99%), and 29.4 g sodium citrate (analytical reagent, ≥ 99%) into 100 mL flask and diluting to the mark with type 1 water, respectively.

### SPE procedure

One millilitre of AG1-X8 resin was placed into a solid-phase extraction tube fitted with a filter (Sigma-Aldrich, 6 mL). After adding the resin, the tube was covered with a second filter, and was connected to an SPE tube adapter and syringe. Throughout the elution process, the flow rate through the SPE was controlled at approximately 2.0 mL/min.

The SPE procedure is outlined below (Table 1). The prepared SPE cartridges were washed with five 1 mL aliquots of type 1 water. Following the washing step water samples prepared with DTPA was applied to the SPE at a flow rate of 2 mL/min. The complex of Tl(III)-DTPA is retained on the resin. Tl(I) is unretained on the SPE resin and is eluted from the cartridge and collected for analysis. The SPE is then washed with type 1 water (5 × 2 mL aliquots) to remove any Tl(I) and the effluent collected and combined with the previous affluent. To elute the Tl(III)-DTPA complex from the column 15 mL of a 0.1 M HCl-SO_2_ solution were loaded on the SPE (5 × 3 mL aliquots) the eluent collected for Tl(III) analysis.

**Table 1:** The SPE procedure for separation of Tl(I) and Tl(III) from environmental aqueous samples

Step	Reagent	Aim
1	5 mL-H_2_O	Cleaning
2	Sample	Introduce the sample (retain Tl(III)-DTPA and leaching Tl(I))
2A	10 mL-H_2_O	Elute the co-retained Tl(I)
3	15 mL-HCl + SO_2_	Elute the Tl(III)

To check the retention of Tl(I) and Tl(III) on AG1-X8 resin, standard solutions of different levels were prepared for SPE by diluting them to desired concentrations with 5 mM DTPA solution. To check the influence of pH, the pHs of standard solutions which contain 3500 ng Tl(I) and 3800 ng Tl(III) were adjusted by adding solutions of nitric acid or sodium hydroxide base on a calibrated pH meter. To check the interferences of complexing ions, wastewater samples were pressure-filtered through a 0.45 μm membrane filter and 100 mL of the 10 mM DTPA solution were added to 100 mL of filtered water, then was divided into two aliquots, one aliquot was spiked by standard solutions of Tl(I) and Tl(III), another without. The two aliquots were treated with the SPE procedure described above on site, the eluents from SPE were collected and acidified for future measurement.

### Chemical analysis

The pH was measured by a pH meter (METTLER TOLEDO, FE20, Zurich, Switzerland). The Tl content was determined by inductively coupled plasma mass spectrometry (ICP-MS) (Agilent, 7700×, California, USA). Recovery of Tl measured in the certified reference material (SLRS-5) was between 94.0% and 102.2% with a relative standard deviation of less than 10%. Rh at 500 μg/L was used as an internal standard and added online.

## Results and discussion

### Retention of Tl(I) and Tl(III) on AG1-X8 resin

When DTPA is added to natural samples containing Tl(III) it forms an anionic group with DTPA, which Tl(I) cannot do. This then enables the separation of Tl(I) and Tl(III) by the anion exchange resin AG1-X8 with the Tl(III)-DTPA complex retaining on the SPE. The results from the recovery of standard solutions (Table 2 and Additional file 1: Table S1) show that almost all Tl(I) is leached from the SPE during the loading of the cartridge and the washing step following the loading. While all added Tl(III) is adsorbed to the SPE resin when Tl(III)-DTPA is added, and no Tl(III) is leached. The recoveries of Tl(I) or Tl(III) were quantitative (100 ± 2% and 99 ± 1% respectively). In real samples, generally Tl(I) is the dominant valence state, thus solutions containing 380 ng Tl(III) and concentrations ratios of 1-, 10-, 50- and 100-fold larger of Tl(I) in 10 mL samples were tested as models of natural aqueous systems. The recoveries of these mixtures of Tl(I) and Tl(III) were satisfactory (98–106%; Table 2 and Additional file 1: Table S1) suggests that the SPE method is fit for the purpose of separating Tl(I) and Tl(III) in water samples.

**Table 2:** Recovery of Tl(I) and Tl(III) from samples prepared by standard solution

Tl species	Tl(I):Tl(III)					
	1:0 (%)	0:1 (%)	1:1 (%)	10:1 (%)	50:1 (%)	100:1 (%)
Tl(I)	100 ± 2	0.2 ± 0.1	101 ± 4	103 ± 6	99 ± 2	97 ± 6
Tl(III)	0.3 ± 0.03	99 ± 1	98 ± 4	100 ± 0.8	106 ± 2	109 ± 1
Total Tl	100 ± 2	99 ± 1	100 ± 4	102 ± 5	99 ± 1	97 ± 6

The samples, all of 10 mL volume, contain 350 ng Tl(I) (1:0 column), 380 ng Tl(III) (0:1 column), or solutions containing 380 ng Tl(III) and concentrations ratios of 1-, 10-, 50- and 100-fold larger of Tl(I) in 10 mL samples were tested. The results are presented as mean values ± SD (n ≥ 3)

To elute Tl(III)-DTPA from the SPE resin, several solutions were assessed, including 6 mol/L HCl, 10 mol/L HNO_3_, 1 mol/L KI, 1 mol/L sodium thiosulfate, 1 mol/L sodium citrate, and 0.1 M HCl-SO_2_ solution. Among them, 6 mol/L HCl and 10 mol/L HNO_3_ did not elute any Tl(III) from the resin, while KI and sodium thiosulfate could precipitate with HNO_3_. The solution of sodium citrate did elute Tl(III)-DTPA from the SPE but lead to difficulties later on when quantifying Tl(III) by ICP-MS. Therefore, the solution of 0.1 M HCl–SO_2_ was selected as the eluent.

### Influence of pH

As pH could influence the hydrolysis of DTPA in water [33, 46], and further adsorption onto the resin, it was essential to establish if pH has an overall effect on the interaction of the Tl(III) and the resin. The results indicate that solutions containing Tl(I) and Tl(III) at various pH’s (from 1 to 7), showed near quantitative recoveries of Tl (95–103%; Table 3 and Additional file 1: Table S2). There was no significant differences in recoveries under different pH conditions according to the Duncan’s new multiple range test base on the SPSS software, therefore, no optimal pH can be established.

**Table 3:** The recovery of Tl(I) and Tl(III) from samples at different pH

pH	Tl(I), %	Tl(III), %	Total Tl, %
pH = 1	102 ± 1	100 ± 6	101 ± 3
pH = 2	98 ± 6	104 ± 6	101 ± 6
pH = 3	97 ± 3	101 ± 2	99 ± 2
pH = 4	95 ± 4	98 ± 0.8	97 ± 2
pH = 5	97 ± 3	99 ± 0.3	98 ± 2
pH = 6	97 ± 2	101 ± 2	99 ± 2
pH = 7	97 ± 0.2	103 ± 3	100 ± 2

The samples at pH from 1 to 7 contain 3500 ng Tl(I) and 3800 ng Tl(III) in 3–8 mL. The results are presented as mean values ± SD (n ≥ 3)

### Standard solution of Tl(III)

As Tl(III) can be easily reduced to Tl(I) under natural conditions, assessing the stability of the Tl(III)-DTPA complex is essential [42]. The preparation process of the standards may be compromised as Tl(III) could be reduced to Tl(I) even in the presence of DTPA. Without oxidation, about 34% of Tl(III) was reduced to Tl(I) in this experiment. Therefore, saturated bromine water was applied for oxidation of Tl(I). Oxidation of Tl(I) was also carried out independently to check the function of DTPA. Saturated bromine water (0.4 mL) was added to a solution containing 4000 ng Tl(I) in 10 mL. The solution was stirred for 15 min with a glass rod and then 10 mL of 10 mmol/L DTPA was added. The solution was stirred for a further 15 min (the color of the solution is yellow), and heated (333 K) until it became colorless and allowed to cool. The resulting solution was then dilution to a total volume of 20 mL and assessed by the SPE method described above. The results showed that when preparing the standard in this way, Tl(I) was fully oxidised to Tl(III), the same results are obtained when replacing DTPA solution with solid DTPA. However, the Tl(III) oxidized by saturated bromine water was quickly reduced to Tl(I) without the addition of DTPA. It should be noted that, although NaOH is frequently used to prepare DTPA solutions [25], DTPA with NaOH is not recommended to treat aqueous samples, as we observed that the addition of NaOH interferes with the oxidation process of Tl(I) to Tl(III) (data not shown).

### Interferences

Natural aqueous samples with elevated Tl are usually characterized by high concentrations of other cations and anions as well, and these other ions could in principle interfere with the analysis. The potential influence of cations on the absorption capacity of the anion exchange resin is limited, however, anions could influence it directly, as they would compete for adsorption onto the anionic group of Tl(III)-DTPA. Thus, two aqueous samples with high concentrations of cations and anions were checked by spiking with standard Tl solutions. The major ions of two aqueous samples are listed in Table 4. Sample A is a leachate sample from Pb–Zn smelting slags in Yunnan Province, China. Sample B is an acid mine drainage (AMD) water sample from Lanmuchang Tl deposits, in Guizhou Province, China. A 1-mL aliquot of the standard Tl solution containing 40 ng of Tl(I) and 40 ng of Tl(III) was added to 1 mL of the two samples, respectively. The average recoveries of Tl(I) and Tl(III) remain identical, at 100 ± 1% (n ≥ 3) and 101 ± 3% (n ≥ 3), respectively, in the two cases, to what had been found in uncontaminated waters.

**Table 4:** Major ions in samples A and B (mg/L)

Sample	Mn	Zn	Cd	Tl	Pb	Fe	Cl^−^	SO_4_^2−^
A	2445 ± 9	5277 ± 13	52 ± 3	0.14 ± 0.02	0.9 ± 0.1	73 ± 6	105 ± 4	21,681 ± 30
B	0.95 ± 0.07	0.4 ± 0.04	< 0.1	0.07 ± 0.002	< 0.1	116 ± 10	0.87 ± 0.03	1283 ± 18

The results are presented as mean values ± SD (n ≥ 3)

### Sample volume

The nominal capacity of AG1-X8 resin is 1.2 meq per mL of resin bed. In theory, 1-mL of AG1-X8 resin can sorb only 1.2/X mmol (X: valence of anion) of anions. The following formula can be used to estimate the volume of sample that can be separated by 1 mL resin:

$${\text{V}}\, = \, 1. 2/\left( {{\text{ax}}\, + \,{\text{by}}\, + \,{\text{cz}}\, + \, \cdots } \right)$$

where the volume of the sample is expressed in V (L), and the concentration of the major anions and the corresponding valence states are expressed by a, b, c, etc.(mmol/L) and x, y, z, etc., respectively. Therefore, to determine the amount of resin needed and the maximum volume of sample that applied to the resin, we need to know the approximate content of the main anions before conducting the experiment. If the total amount of anions in a sample exceeds the capacity of the resin, the adsorbed Tl(III) may be washed off, resulting in a low recovery of Tl(III). For example, the maximum volume of our sample A (Table 4) for 1 mL resin could be estimated by using the concentration of SO_4_^2−^ and Cl^−^ instead of all anions. The maximum volume (V) of sample A for 1 mL resin should be less than 2.6 mL according to the following calculation: V = 1.2/((21,681/96) × 2+(105/35) × 1) = 0.0026 L. Of course, the complex computational process can be replaced by a spike experiment with the same volume of resin and water sample. If the recovery of the spike experiment is quantitative, it establishes that the volume of the sample does not exceed the binding capacity of the resin.

### Suggestion of practical application

The method described has demonstrated an outstanding performance in wastewater samples analyzed from AMD and smelting slags by spiking experiment conducted with Tl(I) and Tl(III) standards as mentioned above. DTPA is necessary for preservation of Tl(III) and determination of Tl speciation. For natural waters, it was found that 0.1 g DTPA per 50 mL samples was required to preserve Tl(III), a similar pretreatment process of water as suggested by Campanella [6]. And it is better to perform spiking experiments with standard solutions of Tl(I) and Tl(III) to check whether interfering compounds of the water samples have an adverse effect on the separation experiment.

The limit of detection (LOD) for Tl species analysis was calculated as 3-times the standard deviation of Tl concentration in the blank samples (10 mL) (mean + 3 × SD, n = 10), and was established as 5 ng/L for Tl(I) and 16 ng/L for Tl(III). DTPA is the only reliable agent for pre-treatment of Tl in water sample, therefore, the present method is less destructive but sufficiently sensitive compare to previous Tl-speciation methods. This method can also separate and pre-concentration of Tl(III) from the dominate Tl(I) and other interfering compounds. Compared to previous SPE methods [25], the resin AG1-X8 is more stable and reliable than alumina.

## Conclusions

The rapid and accurate testing of thallium (Tl) speciation in water samples is important to human and environmental health. In this study, we developed a modified method of solid phase extraction (SPE), using the anion exchange resin AG1-X8, to measure Tl(I) and Tl(III) species in water samples. With the use of diethylene triamine pentacetate acid (DTPA), Tl(III) and Tl(I) species were separated by the formation of Tl(III)-DTPA complex which is selectively adsorbed onto the AG1-X8 resin. The Tl(III)-DTPA can be effectively eluted from the resin with a solution of HCl with SO_2_. The validity of this method was confirmed by the high Tl recoveries of Tl(I) and Tl(III). The present method was demonstrated to have an outstanding performance not only for waters with high Tl(I)/Tl(III) ratios, but also for waters with high levels of interfering compounds, such as SO_4_^2−^, Cl^−^, Zn, Pb, Mn, Fe, and so on. Our method, which can be also used to pre-concentrate Tl(III) and can be performed under wide pH ranges, shows some advantages compared to the commonly used chromatographic method.

## Additional file

**Additional file 1: Table S1.** The measured Tl(I) and Tl(III) from standard solutions at different Tl(I)/Tl(III) ratios. **Table S2.** The measured Tl(I) and Tl(III) from standard solutions at different pH.

## Abbreviations

SPE: solid phase extraction
DTPA: diethylene triamine pentoacetic acid
ICP-MS: inductively coupled plasma mass spectrometry
AMD: acid mine drainage
