# Supplementary material for: A modified method of separating Tl(I) and Tl(III) in aqueous samples using solid phase extraction
Source: Chem Cent J. 2018 Dec 5;12:132. doi: 10.1186/s13065-018-0502-6 (PMC6768029; doi:10.1186/s13065-018-0502-6)
Supplement: Supplementary file 1 — Additional file 1: Table S1. The measured Tl(I) and Tl(III) from standard solutions at different Tl(I)/Tl(III) ratios. Table S2. The measured Tl(I) and Tl(III) from standard solutions at different pH. [file 13065_2018_502_MOESM1_ESM.doc]

**Additional Material**

**A modified method of separating Tl(I) and Tl(III) in aqueous samples using solid phase extraction**

Qingxiang Xiao ^a,c^, Atta Rasool ^a,c^, Tangfu Xiao^b,*^, Philippe C. Baveye ^d^

^a^ State Key Laboratory of Environmental Geochemistry, Institute of Geochemistry, Chinese Academy of Sciences, Guiyang 550081, China. E-mail address: [xiaoqingxiang@mail.gyig.ac.cn](mailto:xiaoqingxiang@mail.gyig.ac.cn).

^b^ Key Laboratory for Water Quality and Conservation of the Pearl River Delta, Ministry of Education; School of Environmental Science and Engineering, Guangzhou University, Guangzhou 510006, China. E-mail address: [tfxiao@gzhu.edu.cn](mailto:tfxiao@gzhu.edu.cn) (T.F. Xiao).

^c^ University of Chinese Academy of Sciences, Beijing 100049, China. E-mail address: [attarasool@vip.gyig.ac.cn](mailto:attarasool@vip.gyig.ac.cn).

^d^ UMR Ecosys, AgroParisTech, Université Paris-Saclay, Avenue Lucien Brétignières, 78850 Thiverval-Grignon, France. E-mail address: [baveye.rpi@gmail.com](mailto:baveye.rpi@gmail.com).

^∗^Corresponding author. Prof. Tangfu Xiao; E-mail address: [tfxiao@gzhu.edu.cn](mailto:tfxiao@gzhu.edu.cn) (T.F. Xiao).

**Running Title:** A modified method of separating Tl(I) and Tl(III) in aqueous samples using solid phase extraction

**Table S1.** **The measured Tl(I) and Tl(III) from standard solutions at different Tl(I)/Tl(III) ratios**

| Tl(I)/Tl(III) | Tl(I) (ng) | |  | | Tl(III) (ng) | |
| --- | --- | --- | --- | --- | --- | --- |
|  | Test | Standard |  | | Test | Standard |
| 1:0 | 349 | 350 | | 1.08 | | 0 |
|  | 343 | 350 | | 1.07 | | 0 |
|  | 355 | 350 | | 0.92 | | 0 |
| 0:1 | 0.67 | 0 | | 379 | | 380 |
|  | 0.24 | 0 | | 377 | | 380 |
|  | 1.31 | 0 | | 370 | | 380 |
| 1:1 | 366 | 350 | | 389 | | 380 |
|  | 340 | 350 | | 362 | | 380 |
|  | 357 | 350 | | 369 | | 380 |
| 10:1 | 3554 | 3500 | | 379 | | 380 |
|  | 3418 | 3500 | | 385 | | 380 |
|  | 3816 | 3500 | | 379 | | 380 |
| 50:1 | 17647 | 17500 | | 396 | | 380 |
|  | 17359 | 17500 | | 406 | | 380 |
|  | 17122 | 17500 | | 409 | | 380 |
| 100:1 | 35109 | 35000 | | 418 | | 380 |
|  | 31567 | 35000 | | 411 | | 380 |
|  | 34712 | 35000 | | 417 | | 380 |

The samples, all of 10 mL volume, contain 350 ng Tl(I) (1:0 column), 380 ng Tl(III) (0:1 column), or solutions containing 380 ng Tl(III) and concentrations ratios of approximately 1-, 10-, 50- and 100-fold larger of Tl(I) (1:1, 10:1, 50:1, and 100:1 columns). Standard: the content of Tl(I) or Tl(III) in the standard solution; Test: the content of Tl(I) or Tl(III) measured by our method. The results are presented as ng.

**Table S2. The measured Tl(I) and Tl(III) from standard solutions at different pH**

| pH | Tl(I) (ng) | |  | | Tl(III) (ng) | |
| --- | --- | --- | --- | --- | --- | --- |
|  | Test | Standard |  | | Test | Standard |
| pH=1 | 3615 | 3500 | | 4028 | | 3800 |
|  | 3586 | 3500 | | 3610 | | 3800 |
|  | 3530 | 3500 | | 3735 | | 3800 |
| pH=2 | 3263 | 3500 | | 3834 | | 3800 |
|  | 3367 | 3500 | | 3787 | | 3800 |
|  | 3694 | 3500 | | 4222 | | 3800 |
| pH=3 | 3443 | 3500 | | 3808 | | 3800 |
|  | 3489 | 3500 | | 3901 | | 3800 |
|  | 3273 | 3500 | | 3749 | | 3800 |
| pH=4 | 3370 | 3500 | | 3759 | | 3800 |
|  | 3434 | 3500 | | 3697 | | 3800 |
|  | 3191 | 3500 | | 3722 | | 3800 |
| pH=5 | 3464 | 3500 | | 3732 | | 3800 |
|  | 3455 | 3500 | | 3747 | | 3800 |
|  | 3261 | 3500 | | 3754 | | 3800 |
| pH=6 | 3421 | 3500 | | 3817 | | 3800 |
|  | 3454 | 3500 | | 3928 | | 3800 |
|  | 3353 | 3500 | | 3805 | | 3800 |
| pH=7 | 3381 | 3500 | | 3832 | | 3800 |
|  | 3383 | 3500 | | 4044 | | 3800 |
|  | 3369 | 3500 | | 3838 | | 3800 |

The samples at pH from 1 to 7 contain 3500 ng Tl(I) and 3800 ng Tl(III) in 3-8 ml. Standard: the content of Tl(I) or Tl(III) in the standard solution; Test: the content of Tl(I) or Tl(III) measured by our method. The results are presented as ng.
